# shinyDSP: a Shiny application for interactive analysis and visualization of NanoString GeoMx Whole Transcriptome Atlas data

**DOI:** 10.1093/bioinformatics/btaf401

**Published:** 2025-07-11

**Authors:** Seung J Kim, Matthew J Cecchini, Marco Mura

**Affiliations:** Interstitial Lung Disease Lab, London Health Sciences Research Institute, London, ON, N6A 5W9, Canada; Department of Pathology and Laboratory Medicine, Schulich School of Medicine and Dentistry, Western University, London, ON, N6A 5C1, Canada; Interstitial Lung Disease Lab, London Health Sciences Research Institute, London, ON, N6A 5W9, Canada; Department of Medicine, Schulich School of Medicine and Dentistry, Western University, London, ON, N6A 5W9, Canada

## Abstract

**Summary:**

NanoString’s GeoMx Digital Spatial Profiling platform enables researchers to elucidate spatial transcriptomic profiles of distinct cellular microenvironments in human and mouse formalin fixed paraffin embedded tissue. To date, there is no free, open source, interactive application to facilitate data analysis with the latest tested methods. We created shinyDSP, a R shiny application that guides users to perform quality control, normalization, and differential gene expression analysis of GeoMx DSP data. It includes various user-provided customization options to meet individual aesthetic choices and requirements.

**Availability and implementation:**

The release and development versions of shinyDSP are available on Bioconductor under the MIT license (https://www.bioconductor.org/packages/release/bioc/html/shinyDSP.html) and Github (https://github.com/kimsjune/shinyDSP).

## 1 Introduction

NanoString’s GeoMx Digital Spatial Profiling (DSP) platform combines gene expression profiling with native tissue architecture (Merritt *et al.* 2020). This represents a significant improvement over traditional bulk RNA-seq, which provides an average gene expression profile across a large population of cells, often obscuring contributions from individual or rare cellular microenvironments within. The workflow begins with a formalin-fixed paraffin embedded (FFPE) section mounted on a glass slide, then stained with fluorescent markers to identify cell populations and tissue architecture. At the same time, mRNA or antibody probes hybridize to their target. These probes are attached to indexed oligonucleotides by a photocleavable linker. Morphological information from immunofluorescence imaging can be integrated into study design and data analysis downstream. Precise regions of interest (ROIs) are defined as any geometric boundary by illuminating the section with UV light, which cleaves the linker and releases the oligonucleotides from probes. These tags are harvested by microcapillary aspiration and counted on the nCounter or sequenced on an Illumina platform. This workflow, combined with the Whole Transcriptome Atlas (WTA) that can quantify over 18 000 and 20 000 genes for human and mouse, respectively, can provide transcriptome-wide spatial gene expression profiles in intact FFPE tissue.

The GeoMx DSP platform’s spatial context stands out in comparison to NanoString’s related nCounter analysis system where fluorescently labelled oligonucleotide barcode-capture probe pairs are used to optically quantify up to 800 targets simultaneously from bulk RNA (Kulkarni 2011). Several data quality control and normalization methods have been proposed for this platform (Waggott *et al.* 2012, Wang *et al.* 2016, Molania *et al.* 2019, Bhattacharya *et al.* 2021). Leveraging these methods, interactive nCounter data processing and analysis applications have been developed, making the platform accessible to users lacking programming experience. For example, NACHO and GUANIN allow users to visualize quality control metrics and apply several normalization schemes (Canouil *et al.* 2020, Montoto-Louzao *et al.* 2024). However, differential gene expression analysis is not available. On the other hand, NanoTube R shiny application enables a simple two-group comparison with limma, gene set enrichment testing and visualization options such as volcano plots, heatmaps and tables (Class *et al.* 2023). However, multiple group comparison is not readily available. Moreover, while output file formats are similar, these applications rely on data analysis methods designed for nCounter, not GeoMx DSP.

Recently, standR has shown robust performance with NanoString GeoMx WTA data compared to existing methods (Liu *et al.* 2024). Briefly, its filtering approach retained more genes with known tissue-specific significance, and implementation of Remove Unwanted Variation 4 (RUV4) normalization corrected slide/patient batch effect. We sought to address the lack of free, open-source, interactive application for analyzing NanoString GeoMx WTA data with the latest tested methods, thus created shinyDSP. Starting from count and annotation tables, shinyDSP can perform quality control with user-provided thresholds, data normalization and create principal component analysis (PCA) plots, fit a linear mixed effects model, identify differentially expressed genes between multiple groups, and render tables, volcano plots and heatmaps as graphical outputs. Throughout these steps, various customization options are available to suit user preferences for data processing and display.

## 2 Key features

- UI elements are dynamically revealed to intuitively guide users through data upload, quality control and visualization.
- Three normalization methods are available.
- A linear mixed effects model with several factors and covariates can be fit to the data (limma-voom).
- Plot aesthetics are highly customizable.
- Plots can be saved into four different file formats (PNG, TIFF, SVG and PDF) to meet most publication requirements.

## 3 Methods and an example use case

The overall workflow diagram is available as Supplementary Data at *Bioinformatics* online. A detailed breakdown of the graphical user interface is available in the vignette on Bioconductor (https://www.bioconductor.org/packages/release/bioc/vignettes/shinyDSP/inst/doc/shinyDSP.html).

### 3.1 Input data and variable selection

**Figure 1.**
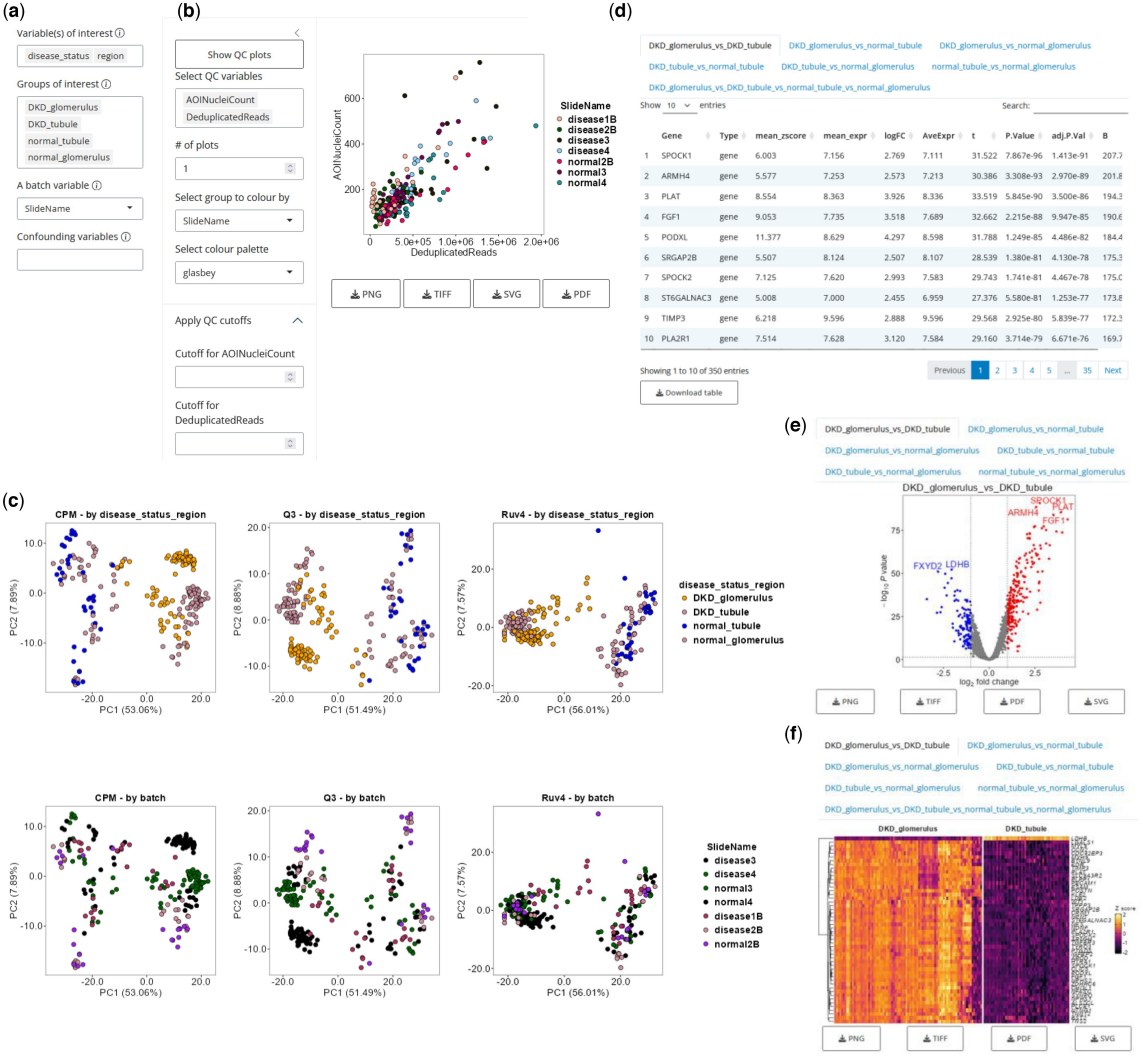
Representative UI and outputs from shinyDSP using the human kidney dataset. (a) Users can select biological variables of interest, sample groups to compare, and block and confounding variables to fit a linear mixed effects model to the data. (b) *X*–*Y* scatter plots can be created with any quantitative categories provided in the annotation table. Any numerical value can be provided as a threshold for filtering. (c) Two sets of PCA plots are generated, colour-coded by biological variables or a block variable. Three normalization methods are shown in each of the three columns. (d) Differentially expressed genes and their statistics are shown in individual panels labelled with groups being compared. (e). Differentially expressed genes between all pairwise comparisons are shown in Volcano plots, separated into tabs. (f) Top differentially expressed genes are shown in heatmaps, separated into tabs.

shinyDSP expects a count and annotation table in either .csv or .txt format as input. These are output files from GeoMx DSP Control Center or ‘readNanoStringGeomxSet()’ function in ‘GeomxTools’ (Griswold *et al.* 2025). To demonstrate the application’s full functionality, the human kidney WTA is included as a sample dataset (Zimmerman *et al.* 2022). Upon loading the demo or real data, the top few rows are displayed on the screen for inspection. If using the demo data, first time users will be prompted to create a new directory called ‘ExperimentHub’ in their R cache folder. Then, users are prompted to identify biological, block and confounding variables automatically parsed from the annotation table. For the kidney dataset, ‘disease_status’ and/or ‘region’ might be biological variables of interest (Fig. 1a). Selecting two or more biological variables concatenates them into one grouped variable. A batch variable is mandatory to account for within-sample correlation as multiple ROIs are sampled from each biological sample in a typical GeoMx WTA dataset. By default, the first column of the annotation table is selected. Lastly, sex, age or sample preparation batch could be confounding variables.

### 3.2 Quality control

To proceed, sample groups, determined by one or more biological variables of interest, must be chosen. For example, selecting ‘disease_status’ and ‘region’ in the kidney dataset allows users to compare between ‘DKD_glomerulus’, ‘DKD_tubule’, ‘normal_tubule’, and ‘normal_glomerulus’. This prompts users to visualize any combination of quantitative variables in x-y scatter plots, and optionally provide a minimum threshold for each variable to filter the original dataset (Fig. 1b). Sample groups can be colour-coded by any categorical variable to reveal any biases in sample quality.

### 3.3 PCA and normalization

Next, three normalization schemes (count-per-million, upper quartile and RUV4) are used to perform PCA and plot the first two components (Fig. 1c). For each method, two plots are generated, one colour-coded by sample groups and the other by the block variable. The latter helps users determine whether there is any subject batch effect, and which normalization method can best correct it. Choosing one of the three normalization methods activates differential gene expression testing using the limma-voom method and prepares three output types: tables, volcano plots and heatmaps (Law *et al.* 2014, Ritchie *et al.* 2015). A detailed explanation of the design matrix is available in the secondary vignette on Bioconductor (https://www.bioconductor.org/packages/release/bioc/vignettes/shinyDSP/inst/doc/shinyDSP_secondary.html).

### 3.4 Tables

Two or more sample groups from 2.2 are required to identify differentially expressed genes. If more than two groups are selected, all possible pairwise comparisons and one-way ANOVA-like F-test across all groups are computed. All differentially expressed genes above the log2 fold change cutoff and below the Benjamini-Hochberg adjusted *P* value (1 and .05 by default, respectively) are reported in a table in tabs labelled with sample groups separated by ‘_vs_’ (Fig. 1d). Tables can be saved as .csv files.

### 3.5 Volcano plots

Similar to the tables, a volcano plot is displayed in tabs labelled with sample groups separated by ‘vs’ for each pairwise comparison (Fig. 1e). Users can provide the following customization options: spot (gene) label density and size, adjusted *P* value and log2 fold change cutoff [to delineate differentially expressed (DE) genes from non-DE genes], colors for up, downregulated, or non-DE genes, and axis range.

### 3.6 Heatmap

Figure 1f shows a sample heatmap of the top 50 differentially expressed genes between ‘DKD_glomerulus’ and ‘DKD_tubule’. By default, hierarchical clustering is applied to the genes (rows) to delineate upregulated and downregulated genes in the two biological groups (columns). The two groups are separated by whitespace. As with tables and volcano plots, heatmaps are separated into tabs. Users can provide the following customization options: the number of genes to show, color palette, Z score range, plot, and gene label size.

### 3.7 Benchmark

A summary table of features across NACHO, GUANIN, NanoTube R shiny, and shinyDSP is available as Supplementary Data at *Bioinformatics* online.

shinyDSP’s underlying R code completes data retrieval, QC, PCA, differential gene expression analysis of the human kidney dataset (∼18 000 genes and ∼200 ROIs across four biological groups) and visualization (volcano plot and heatmap) in about 68 s on a M4 Max Macbook Pro (36GB RAM). A detailed breakdown of execution times for various steps can be found in the secondary vignette on Bioconductor (https://www.bioconductor.org/packages/release/bioc/vignettes/shinyDSP/inst/doc/shinyDSP_secondary.html).

## 4 Conclusion

Overall, shinyDSP offers a complete suite of NanoString DSP WTA data analysis. The application streamlines raw data acquisition to biological interpretation without jeopardizing quality control. The plethora of customization and display options will cater to a wide range of use cases. Future development may include gene set enrichment testing, spatial deconvolution, etc. Issue reports and feature requests can be made on the development branch on Github.

## Supplementary Material

btaf401_Supplementary_Data

## Data Availability

The data underlying this article are publicly available at https://nanostring.com/products/geomx-digital-spatial-profiler/spatial-organ-atlas/human-kidney/. *shinyDSP* also downloads the same data hosted on ExperimentHub.
