# Supplementary figures and images for: shinyDSP: a Shiny application for interactive analysis and visualization of NanoString GeoMx Whole Transcriptome Atlas data
Source: Bioinformatics. 2025 Jul 11;41(7):btaf401. doi: 10.1093/bioinformatics/btaf401 (PMC12311272; doi:10.1093/bioinformatics/btaf401)

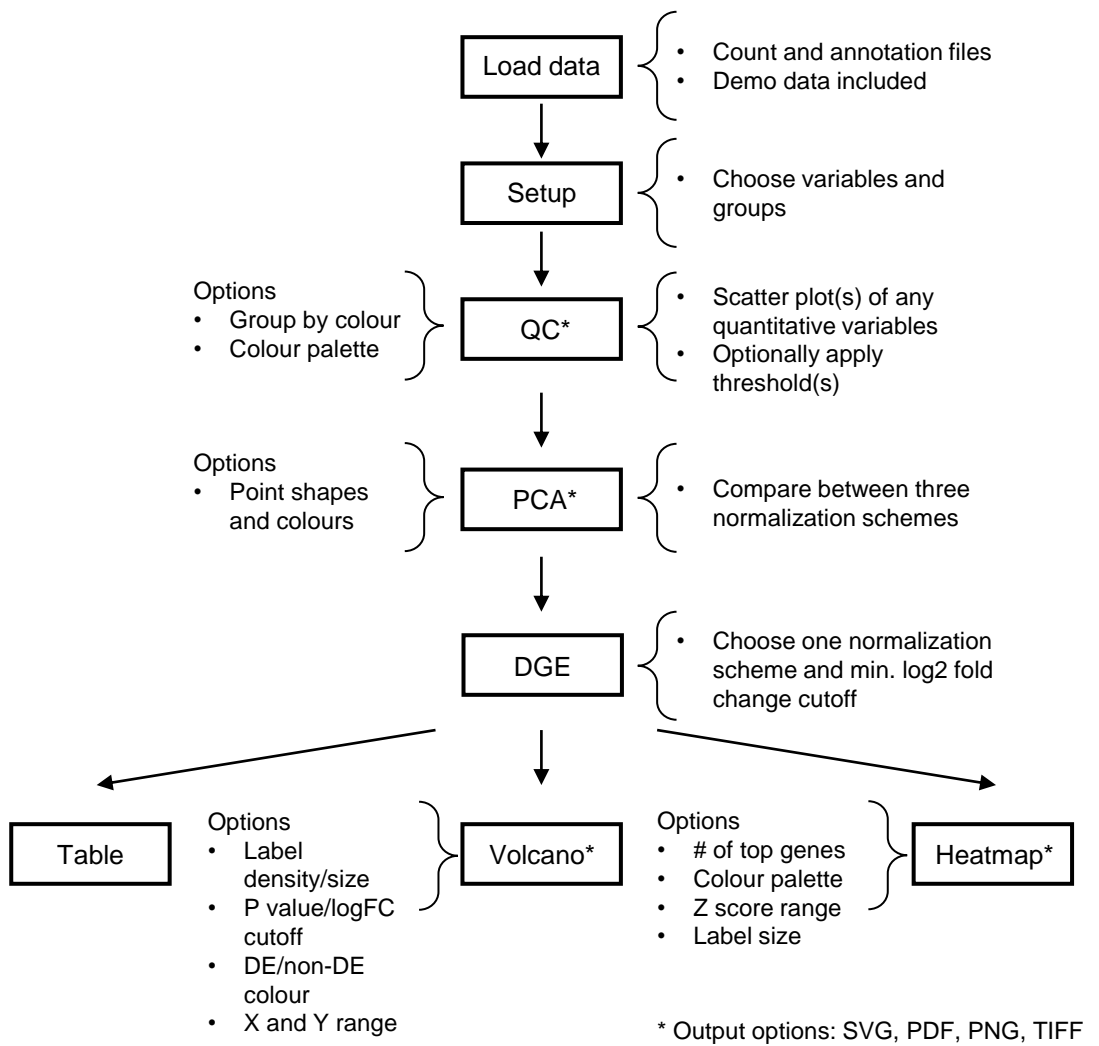

Supplement: btaf401_Supplementary_Data [file btaf401_supplementary_data.zip › SupplementaryData.pdf]
